# Construction and Use of a Recyclable Marker To Examine the Role of Major Facilitator Superfamily Protein Members in *Candida glabrata* Drug Resistance Phenotypes

**DOI:** 10.1128/mSphere.00099-18

**Published:** 2018-03-28

**Authors:** Bao G. Vu, W. Scott Moye-Rowley

**Affiliations:** a Department of Molecular Physiology and Biophysics, Carver College of Medicine, University of Iowa, Iowa City, Iowa, USA; Carnegie Mellon University

**Keywords:** *Candida glabrata*, major facilitator superfamily, selectable marker, transcriptional regulation, transporters

## Abstract

*Candida glabrata* is the second most common species causing candidiasis. C. glabrata can also readily acquire resistance to azole drugs, complicating its treatment. Here we add to the collection of disruption markers to aid in genetic analysis of this yeast. This new construct is marked with a nourseothricin resistance cassette that produces an estrogen-activated form of Cre recombinase in a methionine-regulated manner. This allows eviction and reuse of this cassette in a facile manner. Using this new disruption marker, we have constructed a series of strains lacking different members of the major facilitator superfamily (MFS) of membrane transporter proteins. The presence of 15 MFS proteins that may contribute to drug resistance in C. glabrata placed a premium on development of a marker that could easily be reused to construct multiple gene-disrupted strains. Employing this recyclable marker, we found that loss of the MFS transporter-encoding gene *FLR1* caused a dramatic increase in diamide resistance (as seen before), and deletion of two other MFS-encoding genes did not influence this phenotype. Interestingly, loss of *FLR1* led to an increase in levels of oxidized glutathione, suggesting a possible molecular explanation for this enhanced oxidant sensitivity. We also found that while overproduction of the transcription factor Yap1 could suppress the fluconazole sensitivity caused by loss of the important ATP-binding cassette transporter protein Cdr1, this required the presence of *FLR1*.

**IMPORTANCE** Export of drugs is a problem for chemotherapy of infectious organisms. A class of membrane proteins called the major facilitator superfamily contains a large number of proteins that often elevate drug resistance when overproduced but do not impact this phenotype when the gene is removed. We wondered if this absence of a phenotype for a disruption allele might be due to the redundancy of this group of membrane proteins. We describe the production of an easy-to-use recyclable marker cassette that will allow construction of strains lacking multiple members of the MFS family of transporter proteins.

## Abstract (precis)

Export of drugs is a problem for chemotherapy of infectious organisms. A class of membrane proteins called the major facilitator superfamily contains a large number of proteins that often elevate drug resistance when overproduced but do not impact this phenotype when the gene is removed. We wondered if this absence of a phenotype for a disruption allele might be due to the redundancy of this group of membrane proteins. We describe the production of an easy-to-use recyclable marker cassette that will allow construction of strains lacking multiple members of the MFS family of transporter proteins.

## INTRODUCTION

Invasive candidiasis is the most common nosocomial fungal disease (1). The yeast *Candida glabrata* is the second most common species associated with this disease, likely influenced by its ability to acquire resistance against antifungal therapies (reviewed in reference 2). Infection associated with drug-resistant fungi often leads to poor outcomes and lower survival rates.

C. glabrata typically acquires azole resistance primary through gain-of-function (GOF) mutations in gene encoding a zinc cluster-containing transcription factor called Pdr1 (3–5). These GOF versions of Pdr1 mediate a large transcriptional induction of resistance genes, including the overproduction of an ABCG-class ATP-binding cassette (ABC) transporter-encoding locus, *CDR1*. Studies from many laboratories have demonstrated that the elevated transcription of ABC transporter-encoding genes like *CDR1* is the cause of the enhanced azole resistance of strains containing GOF variants of *PDR1* (4–7). Data also exist that argue these GOF forms of Pdr1 enhance virulence of strains containing these mutant transcription factors (8).

Another class of membrane transporter protein found to be involved in drug resistance includes members of the major facilitator superfamily (MFS) class of proteins (reviewed in reference 9). These proteins are routinely embedded in the plasma membrane and act as drug:H^+^ antiporters (discussed in reference 10). Analysis of the Saccharomyces cerevisiae genome indicated that approximately 22 different MFS proteins thought to be associated with drug resistance can be detected (11). These can further be classed into either drug:H^+^ antiporter (DHA) family 1 or 2, varying by the number of transmembrane domains (12 or 14, respectively) (11, 12). In C. glabrata, 15 members of the DHA1/2 class of MFS proteins can be recognized (13). While the roles of ABC transporters in drug resistance are well accepted, involvement of MFS proteins is less clear. It is relatively common to find that overproduction of MFS proteins elevates drug resistance, but the corresponding gene disruptant often has little effect on this phenotype (discussed in references 14 and 15). One possible explanation for this phenotypic difference is the presence of multiple MFS proteins with redundant physiological roles.

Recent studies in C. glabrata have begun to explore the direct involvement of MFS proteins in azole drug resistance in this pathogenic yeast (16–18). These studies have provided evidence that genes encoding some of these transporter proteins are regulated by Pdr1 (18). Although much of the focus on the membrane proteins impacting drug resistance has centered on ABC transporters, MFS proteins represent a less studied class of resistance determinants that are very likely to contribute to this phenotype in an important manner.

Specifically in the case of C. glabrata, the presence of 15 MFS proteins that may influence drug resistance provides a relatively complicated genetic background to assess individual contributions of each membrane transporter. Experience in S. cerevisiae has also demonstrated unexpected interactions of amino acid auxotrophies with membrane trafficking pathways and even ABC transporter proteins (19, 20). To facilitate analyses of MFS proteins in drug resistance, we wanted to develop a dominant disruption marker that could be used in clinical isolates as well as laboratory prototrophic strains. We also wanted to be able to repetitively use this marker cassette in order to generate strains lacking multiple members of the MFS protein family in case redundancy of this group of proteins might mask phenotypes caused by loss of a small number of these proteins.

In this study, we constructed a tightly regulated and inducible recyclable construct for gene disruption and insertion in C. glabrata. This construct takes advantages of the Cre-loxP-based recombination, which has been used extensively in a wide variety of genetic systems to remove chromosome segments of interest. To control the levels of expression and activity of Cre, the methionine-regulatable promoter (*MET3*) is used to drive transcription of the *cre* gene and Cre activity is posttranscriptionally regulated by fusion to the estradiol-binding domain (EBD) of the murine estrogen receptor. The EBD sequesters the fusion protein in the cytoplasm until estradiol is introduced, at which point the fusion protein is transported into the nucleus where Cre can act upon its loxP DNA substrates. Together, Cre activity is tightly controlled by methionine and can also be conditionally induced with extracellular estradiol. Further applying our new *loxP-cre*-EBD recyclable system into C. glabrata genetics, we also found that C. glabrata* CAGL0H06017g*, a homolog of S. cerevisiae* FLR1*, has a significant role in oxidative stress and fluconazole resistance of the organism.

## RESULTS

### Assembling a recyclable construct.

**FIG 1:**
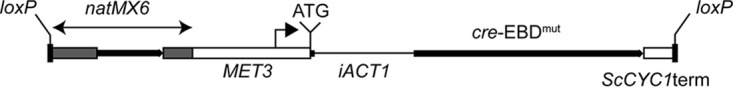
Structure of the evictable marker cassette. A diagram of the recyclable cassette containing the nourseothricin resistance marker (*natMX6*) is shown. The two *loxP* recombination targeting sites are indicated by small black bars at the ends of the cassette. The location of the methionine-repressible MET3 promoter is shown with its direction of transcription indicated by the arrow. The position of the *cre*-EBD^mut^ fusion gene is shown by the solid black arrow with the location of the inserted C. glabrata actin intron denoted as *iACT1*. Transcription termination is provided by the relevant region of the S. cerevisiae* CYC1* gene (*ScCYC1*term).

A diagram of the recyclable marker cassette is shown in Fig. 1. This construct was designated pBV65. The key features of this plasmid center on its modified *cre* gene with flanking *loxP* repeats. We based our construct on work from Lindstrom and Gottschling, who utilized selection in S. cerevisiae to isolate a highly estrogen-responsive form of a Cre-EBD fusion protein (21). Our initial cloning efforts failed as even the low activity of this Cre-EBD fusion protein in bacterial cells prevented recovery of the desired full-length clone. To further lower any undesired bacterial activity of Cre-EBD, we inserted an intron from the C. glabrata* ACT1* gene into the amino-terminal-coding region of the *cre*-EBD gene. This new construct was stable in bacteria and allowed us to isolate and characterize the desired recyclable marker cassette. A similar strategy has been used before in Candida albicans (22).

We also placed the *cre*-EBD gene under control of the methionine-repressible C. glabrata* MET3* promoter (23). In this manner, we would limit transcription of this gene by including methionine in the medium and limit the activity of whatever Cre-EBD protein that might be produced with the exclusion of any added estrogen. This original marker cassette included a codon-optimized form of the nourseothricin resistance gene under control of the *TEF2* promoter/terminator from Ashbya gossypii. We also prepared two related plasmids varying only in the marker. A Saccharomyces kluveryi *HIS3* or S. cerevisiae *URA3* marker replaced the *natMX6* cassette in these two related constructs. Together, the dominant *natMX6* marker coupled with the *HIS3* and *URA3* markers should permit wide usage of these cassettes in different genetic backgrounds.

### Testing the recyclable construct in CBS138.

We initially used the pBV65 plasmid to produce a disruption allele that would target the *CDR1* ABC transporter-encoding gene. Mutants lacking Cdr1 are well known to exhibit a profound fluconazole-hypersensitive phenotype (24). PCR was used to prepare 800-bp fragments from the immediate upstream and downstream regions of the *CDR1* gene. These fragments were then assembled along with the marker cassette from pBV65 into a pUC19 vector plasmid. After confirmation of the structure of this plasmid (pBV65), we digested this construct with restriction enzymes to release the vector and transformed the resulting digest into competent CBS138 cells. Transformants were selected on rich yeast extract-peptone-dextrose (YPD) medium containing nourseothricin and methionine. We used CBS138 as the recipient strain since this is the standard sequenced C. glabrata strain and is prototrophic. (The strains used are listed in Table 1.)

**TABLE 1:** Strains used in this study

Strain	Relevant genotype
CBS138	Wild type
BVG27	*cdr1*Δ::*loxP*
BVG65	*pdr1*Δ::*loxP*
BVG41	*flr1*Δ::*loxP*
BVG39	*aqr1*Δ::*loxP*
BVG47	*qdr2*Δ::*loxP*
BVG45	*aqr1*Δ::*loxP flr1*Δ::*loxP*
BVG43	*aqr1*Δ::*loxP qdr2*Δ::*loxP*
BVG49	*flr1*Δ::*loxP qdr2*Δ::*loxP*
BVG51	*aqr1*Δ::*loxP flr1*Δ::*loxP qdr2*Δ::*loxP*
BVG67	*TDH3-YAP1*
BVG69	*TDH3-YAP1 cdr1*Δ::*loxP*
BVG71	*TDH3-YAP1 pdr1*Δ::*loxP*
BVG77	*TDH3-YAP1 flr1*Δ::*loxP*
BVG73	*TDH3-YAP1 aqr1*Δ::*loxP flr1*Δ::*loxP qdr2*Δ::*loxP*
BVG100	*TDH3-YAP1 cdr1*Δ::*loxP flr1*Δ::*loxP*

**FIG 2:**
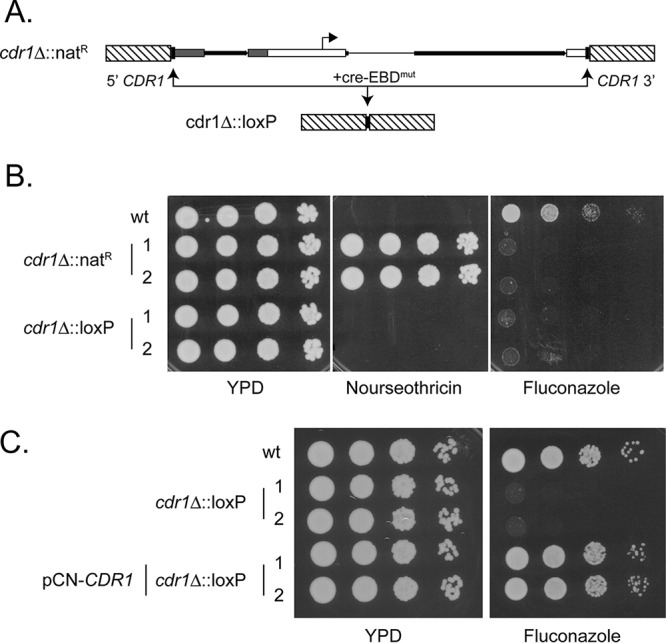
Construction of a *cdr1*Δ::*loxP* allele using the new recyclable marker cassette. (A) Diagram showing the plasmid construct containing the *cdr1*Δ::*loxP* allele that was introduced into CBS138 cells with selection for nourseothricin resistance (*nat*^R^). This allele removes the entire *CDR1* open reading frame (ORF). The result of recombination between the two *loxP* repeats is indicated by the arrow resolving to a single repeat denoted by the small black box. (B) Phenotypes of primary disruption mutants and evicted counterparts. Isogenic wild-type (CBS138), primary disruptants (*cdr1*Δ::*nat*^R^), and evicted disruptants (*cdr1*Δ::*loxP*) were grown to mid-log phase, and then serial dilutions were spotted across the plates. Two independent isolates of each disruptant were tested. (C) Complementation of the fluconazole-hypersensitive phenotype of the *cdr1*Δ::*loxP* allele by a plasmid-borne copy of *CDR1*. Wild-type and *cdr1*Δ::*loxP* cells were transformed with an empty vector containing the nourseothricin resistance gene and served as controls for this experiment. The *cdr1*Δ::*loxP* cells were also transformed with the same vector plasmid containing a wild-type version of the *CDR1* gene. The indicated transformants were serially diluted on rich YPD medium lacking or containing 5 μg/ml fluconazole (as well as 50 μg/ml nourseothricin to select for plasmid maintenance).

Nourseothricin-resistant clones were recovered, and the structure of the integrated marker cassette was verified as described in the legend to Fig. 2A. To excise the marker cassette, two independent clones were grown in minimal medium lacking methionine and supplemented with 1 μM estradiol overnight. Single colonies were collected and subsequently tested for nourseothricin sensitivity. The absence of the cassette on the chromosome was examined by PCR. Finally, primers located outside the recombined regions were used to amplify across the *CDR1* coding region to confirm the complete removal of the gene (Fig. 2A).

Along with characterization of the genotype of these *cdr1*Δ disruption mutants, we confirmed the expected resistance phenotypes of each derivative. We grew CBS138 cells along with the primary nourseothricin-resistant and evicted transformants in YPD medium and tested these clones for resistance to either nourseothricin or fluconazole. Mid-log-phase cells were serially diluted and placed on medium containing either 50 μg/ml nourseothricin or 5 μg/ml fluconazole. These plates were incubated at 37°C and then photographed (Fig. 2B).

Only the primary transformants grow on nourseothricin-containing medium, while only the wild-type CBS138 cells can grow on fluconazole-containing medium. We also prepared a low-copy-number plasmid marked with a nourseothricin resistance gene that carried a wild-type version of the *CDR1* gene. Introduction of this plasmid into independent *cdr1*Δ::*loxP* cells restored fluconazole resistance to normal levels (Fig. 2C).

We also assessed the stability of the *cre*-EBD cassette under various medium conditions. A nourseothricin-resistant transformant was grown overnight using different medium formulations. The next morning, cells were plated on rich YPD medium or YPD containing nourseothricin. The plating efficiency of these cells was determined by dividing the number of colonies formed on nourseothricin-containing medium by the number grown on YPD medium alone (Table 2).

**TABLE 2:** Dependence of recycling probability on medium

Medium	Recycling probability (%)^a^	SD (%)
YPD	0	5
YPD + Met	0	10
YPD + Met + E2	15	4
YPD + E2	46	9
CSM + Met	5	4
CSM + Met + E2	11	12
CSM	18	8
CSM + E2	98	1

a Percentage of nourseothricin-sensitive colonies from total colonies. This was determined by comparing the plating efficiency of cells grown overnight on the indicated medium, followed by plating on YPD or YPD plus nourseothricin.

Overnight growth on minimal medium lacking methionine and containing estrogen led to a nearly 100% loss of the *natMX6* marker, consistent with the high efficiency of Cre-EBD-driven recombination under this medium condition. The presence of estrogen was the most efficient inducer of recombination as addition of this steroid to YPD alone led to nearly 50% loss of the *natMX6* marker. We interpret these data to indicate that in the absence of methionine and estrogen, the *natMX6* marker in this cassette is highly stable.

### Multiple MFS genes knocked out mutants in CBS138.

**FIG 3:**
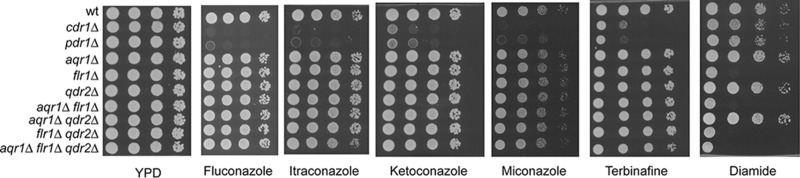
Phenotype profiling of isogenic CBS138 *aqr1*, *flr1*, and *qdr2* deletion mutants. Serial dilutions of the indicated isogenic derivatives of CBS138 (wild type [wt]) were placed on these various media. All drugs were added to YPD at the following concentrations: 5 µg/ml fluconazole, 0.2 µg/ml itraconazole, 0.2 µg/ml ketoconazole, 2 µg/ml miconazole, 20 µg/ml terbinafine, and 3 mM diamide.

Construction of the *cdr1*Δ::*loxP* mutant served as a positive control for the utility of this recyclable marker cassette. We next constructed three different disruption alleles using this recyclable cassette in different MFS protein-encoding genes. We selected the *AQR1* (16, 25), *FLR1* (26), and *QDR2* (18) loci to disrupt based on the work of others that has linked these MFS proteins to resistance phenotypes in C. glabrata. One of the potential complications in the analysis of the physiological roles of MFS proteins in general, and in C. glabrata in particular, is the possible redundancy in function provided by the typically large number of these related membrane transporters. To begin to examine functional overlap between MFS proteins involved in drug resistance, we utilized the recyclable marker system described above to generate single, double, and triple mutant strains lacking these selected genes. We also compared the phenotypes of these MFS deletion strains with the *cdr1*Δ::*loxP* strain described above as well as a similarly constructed *pdr1*Δ::*loxP* strain lacking the Pdr1 transcription factor that is well established to exhibit drug-sensitive phenotypes (3). These strains were all grown to mid-log phase and then serially diluted on plates containing various concentrations of the azole drugs, terbinafine or diamide (Fig. 3A).

While loss of either *PDR1* or *CDR1* led to strong azole- and terbinafine-sensitive phenotypes, we were unable to detect significant defects in resistance to either of these drugs upon loss of any combination of the selected MFS proteins. We did find pronounced diamide hypersensitivity in the presence of the *flr1*Δ::*loxP* allele, as described before. Removal of the other MFS proteins from the *flr1*Δ::*loxP* background did not lead to further exacerbation of diamide sensitivity, indicating this phenotype was unique to Flr1 in our strains.

### *FLR1* is essential for C. glabrata resistance to diamide-induced oxidative stress.

**FIG 4:**
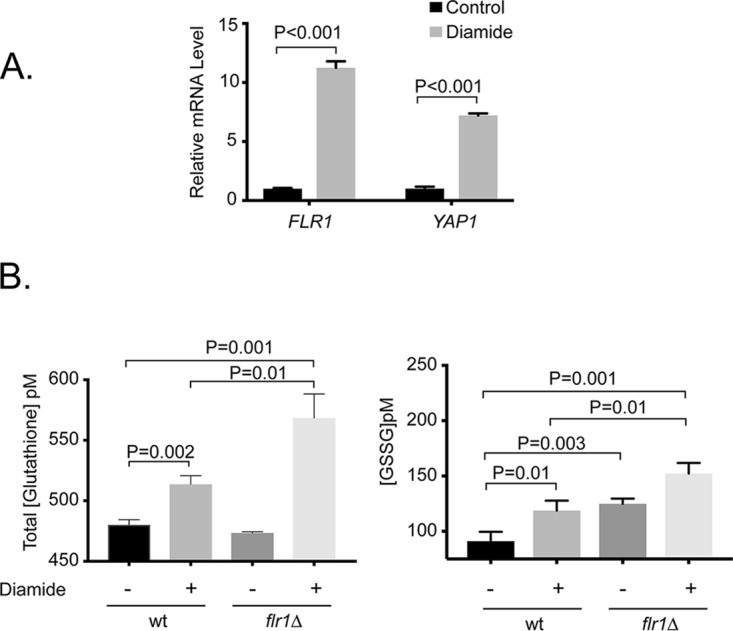
*FLR1* expression and glutathione homeostasis. (A) Induction of *FLR1* and *YAP1* mRNA upon diamide challenge. Wild-type (wt) cells were grown to mid-log phase and then treated with 4 mM diamide or left untreated for 20 additional min. Levels of mRNA were assayed using RT-qPCR. (B) Glutathione levels in wild-type and *flr1*Δ mutant cells in response to diamide. A commercial kit was used to measure total glutathione (reduced plus oxidized) as well as the oxidized form (GSSG) in wild-type and isogenic *flr1*Δ cells in the presence or absence of diamide.

While previous work had implicated *FLR1* in diamide tolerance (26), the molecular details underlying this phenotype remain unknown. Work from S. cerevisiae has established that S. cerevisiae* FLR1* (*ScFLR1*) is transcriptionally induced by *Sc*Yap1 upon exposure to diamide (27). We tested if Yap1 had a similar relationship with *FLR1* transcription in C. glabrata by measuring *FLR1* and *YAP1* mRNA levels before and after diamide stress using a reverse transcriptase quantitative PCR (RT-qPCR) assay (Fig. 4A). These data confirm that *FLR1* was induced in response to diamide but also that *YAP1* transcription was similarly elevated. The induction of *YAP1* mRNA by diamide was not expected as extensive studies in S. cerevisiae indicate that this factor responds posttranslationally to diamide challenge by regulation of its nuclear export (28, 29).

Diamide causes an increase in the levels of oxidized glutathione (GSSG) in cells (30). Since glutathione (GSH) is the primary reducing agent in the cell, it is this depletion of reduced glutathione that is thought to underly the toxicity of diamide (30). We measured total and oxidized glutathione (Fig. 4B) levels in isogenic wild-type and *flr1*Δ::*loxP* strains before and after diamide challenge. Total glutathione levels in these strains were the same prior to diamide challenge. The *flr1*Δ::*loxP* strain displayed a significant increase in glutathione levels following diamide stress compared to isogenic wild-type cells. Strikingly, the levels of oxidized glutathione were the same in diamide-stressed wild-type cells and the unchallenged *flr1*Δ::*loxP* strain. This argues that loss of Flr1 caused a defect in glutathione homeostasis even in cells grown in rich YPD medium. Treatment of *flr1*Δ::*loxP* cells caused an additional elevation in oxidized glutathione. These data are consistent with Flr1 being required for maintenance of normal cellular glutathione levels.

### Overexpression of *YAP1* induced the expression of *FLR1* and diamide resistance.

**FIG 5:**
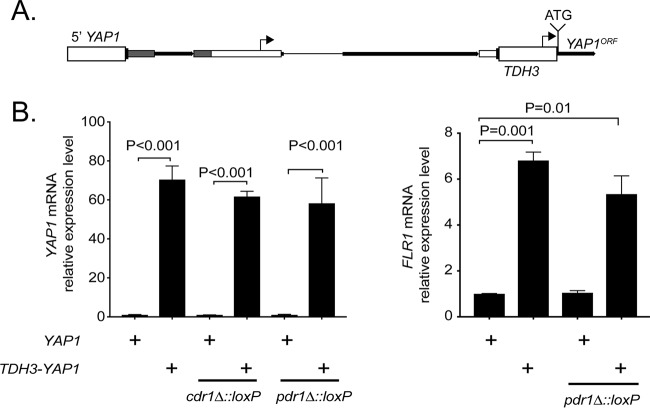
Overexpression of *YAP1* induces the expression of *FLR1*. (A) Use of the recyclable cassette to replace the *YAP1* promoter with the strong glycolytic *TDH3* promoter in the chromosome. (B) RT-qPCR measurement of the induction of *YAP1* mRNA when driven by the *TDH3* promoter versus wild-type *YAP1* regulatory sequences. Wild-type, *pdr1*Δ::*loxP*, and *cdr1*Δ::*loxP* strains with native or *TDH3-YAP1* alleles were used for these mRNA determinations. (C) Assay for *FLR1* mRNA levels by RT-qPCR in the indicated strains.

The findings above indicating that transcription of both *YAP1* and *FLR1* was induced upon diamide exposure led us to test the effect of Yap1 overproduction on phenotypes involving *FLR1*. We adapted the recyclable marker for use as an overexpression cassette by placing this maker upstream of the C. glabrata* TDH3* promoter. *TDH3* encodes the highly expressed enzyme glyceraldehyde-3-phosphate dehydrogenase (GAPDH). A cassette was generated with this recyclable marker-*TDH3* promoter cassette placed between the *YAP1* promoter and the ATG for the *YAP1* open reading frame (Fig. 5A). This promoter insertion fragment was transformed into isogenic wild-type, *cdr1*Δ::*loxP*, *pdr1*Δ::*loxP*, *flr1*Δ::*loxP*, *cdr1*Δ::*loxP flr1*Δ::*loxP* double mutant, and *aqr1*Δ::*loxP flr1*Δ::*loxP qdr2*Δ::*loxP* triple mutant strains with selection for the nourseothricin cassette. We selected transformants that showed the correct genotype, evicted the recyclable marker, and then used these *TDH3* promoter-driven *YAP1* strains to test diamide and fluconazole resistance in appropriate transformants.

We first validated that the replacement of the standard *YAP1* promoter with the *TDH3* promoter fragment would lead to increased *YAP1* mRNA levels by using RT-qPCR (Fig. 5B). The *TDH3* promoter did lead to markedly elevated *YAP1* mRNA production (~60× higher than wild type) in all strains tested. We also examined the response of *FLR1* to overproduced Yap1 and found roughly a 6-fold increase in *FLR1* mRNA when Yap1 was expressed from the *TDH3* promoter.

**FIG 6:**
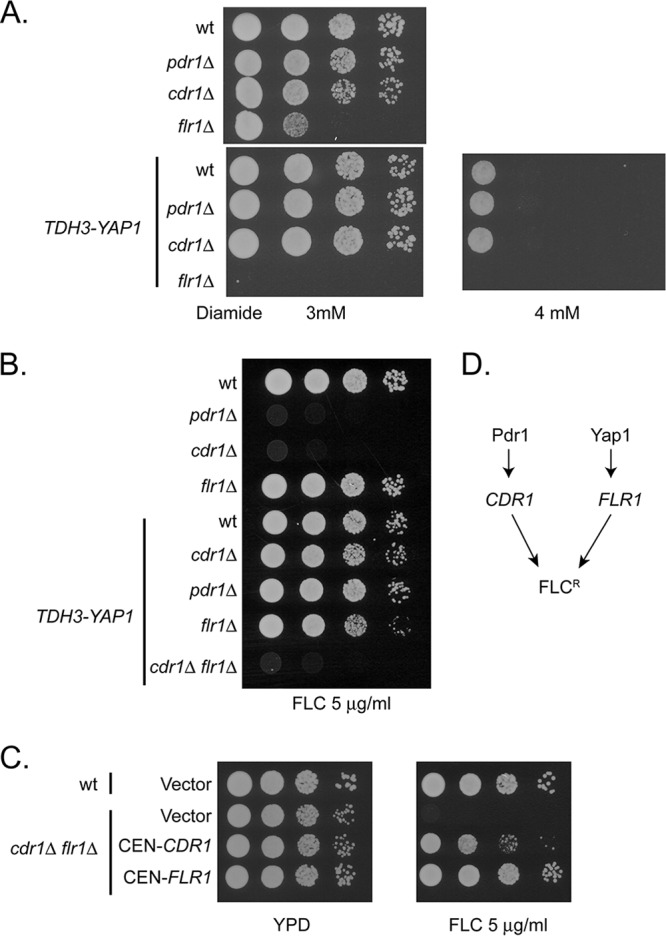
Interaction of Yap1- and Pdr1/Cdr1-regulated resistance pathways. (A) *TDH3-YAP1* drives elevated diamide resistance independent of *PDR1* or *CDR1*. Isogenic wild-type, *pdr1*Δ::*loxP*, and *cdr1*Δ::*loxP* strains containing or lacking the *TDH3-YAP1* allele were grown to mid-log phase and then serially diluted on plates containing the indicated concentrations of diamide. (B) The indicated strains were grown to mid-log phase and then serially diluted on rich medium containing 5 μg/ml fluconazole (FLC). (C) Complementation of fluconazole sensitivity in a *cdr1*Δ* flr1*Δ double mutant strain. Isogenic wild-type or *cdr1*Δ* flr1*Δ mutant strains were transformed with the plasmids indicated at left, which are all low-copy-number CEN-containing vectors with nourseothricin resistance. Both plates contain low-level nourseothricin (50 μg/ml) to ensure retention of the plasmids. (D) Diagram of interactions relating Pdr1/Cdr1 to Yap1/Flr1 and azole resistance. Arrows indicate positive interactions.

Having determined that the *TDH3-YAP1* fusion gene was having the expected effect on both *YAP1* and *FLR1* mRNA levels, we then compared the resistance phenotypes of strains varying in their expression of Yap1. Overproduction of Yap1 clearly elevated diamide resistance in all backgrounds tested, except in the absence of *FLR1*, which blocked this effect (Fig. 6A). The Yap1-dependent elevation of diamide required the presence of *FLR1*, suggesting that induction of *FLR1* transcription led to this oxidant resistance phenotype.

Earlier work in both S. cerevisiae and C. glabrata found that loss of the multidrug resistance pathway defined by *Sc*Pdr5 or C. glabrata Cdr1 (*Cg*Cdr1) or the transcription factor Pdr1 (in either yeast) caused a profound drug-sensitive phenotype (recently reviewed in reference 31). Epistasis experiments with overproduction of *YAP1* in S. cerevisiae and later C. glabrata also indicated that high levels of Yap1 could influence drug resistance and even bypass the drug sensitivity of a *pdr5*Δ allele in S. cerevisiae (32). To evaluate the epistasis of Yap1 and *PDR1/CDR1* in C. glabrata, we compared the fluconazole phenotypes of isogenic wild-type and *pdr1*Δ::*loxP*, *cdr1*Δ::*loxP*, and *flr1*Δ::*loxP* single mutant strains or a *cdr1*Δ::*loxP flr1*Δ::*loxP* double mutant containing wild-type *YAP1* or the *TDH3-YAP1* fusion gene. These strains were all tested by serial dilution for their ability to grow on fluconazole-containing medium.

Loss of either *PDR1* or *CDR1* led to the expected decrease in fluconazole resistance (Fig. 6B). Similarly to S. cerevisiae (32), overproduction of Yap1 in these backgrounds returned resistance to near wild-type levels at this drug concentration. However, removal of the *FLR1* gene from the *cdr1Δ*::*loxP TDH3-YAP1* strain abolished this suppression of the fluconazole sensitivity caused by loss of *CDR1*. These data are most consistent with Yap1 driving expression of *FLR1* with a corresponding increase in azole resistance acting in a parallel fashion to the Pdr1-*CDR1* circuit (Fig. 6D).

We used plasmid complementation to ensure that *FLR1* gene activation by Yap1 was responsible for the bypass of fluconazole sensitivity of the *cdr1*Δ::*loxP* strain. The *cdr1*Δ::*loxP flr1*Δ::*loxP* double mutant carrying the *TDH3-YAP1* gene was transformed with low-copy-number plasmids containing wild-type copies of either *CDR1* or *FLR1*. Appropriate transformants were then tested for fluconazole resistance (Fig. 6C).

Reintroduction of either *CDR1* or *FLR1* on a low-copy-number plasmid elevated the fluconazole of the resulting transformants back to levels similar to those in the wild-type strain containing the *TDH3-YAP1* fusion gene. Complementation by the CEN-*CDR1* clone was slightly lower than expected, possibly due to differential stability or copy number of the plasmid vector. The exquisite sensitivity of the *cdr1*Δ *flr1*Δ double mutant strain is due to loss of both of these two key target genes that would otherwise elevate fluconazole resistance.

## DISCUSSION

A central goal of this work was to develop a system in which repetitive gene disruptions could easily be performed as our aim is ultimately to construct strains lacking multiple members of the MFS protein-encoding genes. A second criterion was that a dominant marker needed to be used so that we could carry out experiments in clinical isolates that are routinely prototrophic. We believe that this new nourseothricin resistance-marked recyclable cassette provides such a facile system. Our incorporation of the estrogen-regulated Cre protein along with the transcriptional control of the methionine-repressible *MET3* promoter keeps the nourseothricin resistance marker in a stable form until eviction is desired. Addition of estrogen and growth in medium lacking methionine led to nearly 100% loss of the nourseothricin resistance marker, allowing the cassette to be used again. The availability of *HIS3*- and *URA3*-marked derivatives will facilitate use of this cassette in laboratory strains containing appropriate auxotrophies.

We also developed a promoter fusion cassette in which the C. glabrata* TDH3* promoter is placed immediately adjacent to this recyclable marker. This allowed us here to place the *YAP1* gene under control of the *TDH3* promoter, one of the strongest in C. glabrata (33). The *TDH3-YAP1* fusion gene overproduced *YAP1* mRNA by approximately 60-fold compared to the wild-type *YAP1* promoter. Future derivatives will be constructed to include an amino-terminal epitope tag to allow direct visualization of the levels of protein production. We relied only on mRNA measurements to argue for overproduction of Yap1, but the large extent of mRNA induction and increased resistance phenotypes suggest that the protein is overproduced in strains carrying this gene fusion.

We found that overproduction of Yap1 was able to strongly suppress the fluconazole sensitivity of either a *pdr1*Δ or *cdr1*Δ strain. This suppression was completely dependent on the presence of *FLR1*. These data resemble findings in S. cerevisiae in which overproduction of Yap1 fully suppressed the drug sensitivity of a *pdr5*Δ strain (32). Overproduction of Yap1 in S. cerevisiae strongly elevated drug resistance in wild-type cells, but we found only minor effects on the drugs tested here in C. glabrata transformants containing *TDH3-YAP1* (data not shown). However, the profound drug sensitivity caused by loss of either *CDR1* or *PDR1* was strongly suppressed by Yap1 overproduction in both yeasts. These data indicate that the parallel pathway to drug resistance defined by Yap1 and *FLR1* still plays an important role in C. glabrata but appears to be a more minor contributor than the Pdr1-*CDR1* circuit.

A remarkable effect occurring upon loss of Flr1 was seen in our glutathione measurements. Deletion of *FLR1* triggered an increase in levels of oxidized glutathione even in the absence of any oxidative stress (Fig. 4C). This represents a selective alteration in the glutathione pool as the total level of glutathione (reduced plus oxidized) is unchanged in isogenic wild-type and *flr1*Δ strains (Fig. 4B). These data suggest that Flr1 may act to either maintain the normal oxidative environment in the cell or to support export of oxidized glutathione from the cell. Changes in glutathione homeostasis have been linked to antifungal activity by others (34). Previous studies in S. cerevisiae have identified ABC transporters like Yor1 as well as MFS family members like Gex1/2 as being involved in export of reduced glutathione to detoxify arsenic-related compounds (35, 36). The role of Flr1 in glutathione homeostasis could also be involved in the influence of this protein on drug resistance as glutathione conjugation can be used as a means of detoxification of harmful chemicals (reviewed in reference 37). Loss of the proper forms of glutathione could interfere with appropriate drug detoxification. This was seen previously in S. cerevisiae, in which loss of glutathione transferase genes caused sensitivity to diamide-induced oxidative stress (38). Similarly, experiments in the fission yeast Schizosaccharomyces pombe identified glutathione transferase enzymes as being required for normal resistance to fluconazole (39), again directly linking glutathione metabolism to drug resistance.

As has been seen before in S. cerevisiae (32) and C. albicans (40), our data argue that Yap1 in C. glabrata plays a role in drug resistance that involves control of MFS protein-encoding gene expression acting in parallel to regulation of genes encoding ABC transporter gene expression. In all three of these yeasts, zinc cluster transcription factors (Pdr1/3 in S. cerevisiae, Tac1 in C. albicans, and Pdr1 in C. glabrata) primarily provide transcriptional regulation of the expression of ABC transporters. Disruption of the primary ABC transporter target gene in any of these yeasts (*PDR5* in S. cerevisiae or *CDR1* in the *Candida* species) led to profound drug sensitivity. While Yap1 (Cap1 in C. albicans) is routinely associated with MFS transporter-encoding gene expression, loss of these genes does not typically elicit drug sensitivity. The striking conservation of these parallel avenues impacting drug resistance suggests an equally important underlying cell physiology maintained in these yeasts. Understanding these conserved regulatory networks for ABC and MFS transporter-encoding gene expression is an important goal in extending our understanding of fungal drug resistance.

## MATERIALS AND METHODS

### Media, plasmids, and strains.

Unless otherwise specified, cells were grown in rich YPD medium (1% yeast extract, 2% peptone, 2% glucose) or under amino acid selective conditions in complete supplemental medium, (CSM: Difco yeast nitrogen extract without amino acids, amino acid powder from Sunrise Science Products, 2% glucose). All strains used in this study are listed in Table 1. Three new plasmids were constructed for this work. The first is pCN, a nourseothricin-resistant vector plasmid produced from a previously constructed low-copy-number plasmid (pSKB60) by Gibson assembly of a *natMX6* cassette in place of the original *ScLEU2* gene. This plasmid is referred to as pBV133. Two derivatives of pBV133 containing either the wild-type *CDR1* gene (pBV201) or the wild-type *FLR1* gene (pBV193) were then produced by Gibson assembly of appropriate fragments into this nourseothricin-resistant vector backbone. The *CDR1* gene fragment contained 1.5 kb of 5′ and 1 kb of 3′ noncoding DNA, while the *FLR1* gene fragment contained 1 kb of 5′ and 0.3 kb of 3′ noncoding DNA. The plasmid used to replace the *YAP1* promoter with the *TDH3* promoter was designated pBV163. These plasmids were confirmed by restriction mapping and DNA sequencing.

### Drug sensitivity assay.

Cells were grown in YPD to mid-log-phase cultures. Then, 10^3^ cells were spotted onto YPD plates containing 5 µg/ml fluconazole (LKT Labs, St. Paul, MN), 0.2 µg/ml itraconazole (LKT labs, St. Paul, MN), 0.2 µg/ml ketoconazole (Sigma-Aldrich, St. Louis, MO), 2 µg/ml miconazole (Sigma-Aldrich, St. Louis, MO), 20 µg/ml terbinafine (Sigma-Aldrich, St. Louis, MO), or 3 or 4 mM diamide (Sigma-Aldrich, St. Louis, MO). Plates were incubated at 37°C for 24 h before imaging.

### *C. glabrata* transformation and recycling marker.

Cell transformations were performed using a lithium acetate method (41). After being heat shocked, cells were grown in YPD at 30°C at 200 rpm overnight and plated on YPD agar plates supplemented with 2 mM methionine (Fisher Scientific, Chicago, IL) and 50 µg/ml of nourseothricin (Jena Bioscience, Jena, Germany). Plates were incubated at 30°C for 24 to 48 h before individual colonies were isolated and screened by PCR for correct insertion of the targeted construct. To induce Cre-dependent recombination, selected clones were grown in methionine-deficient synthetic complete (SC) medium supplemented with 1 µM estradiol (Sigma-Aldrich, St. Louis, MO) at 30°C at 200 rpm overnight. Single colonies were streaked onto YPD plates and incubated at 30°C overnight. Colonies were screened for the loss of nourseothricin resistance, and sensitive colonies were selected for further experiments.

### Measurement of *in vivo* glutathione level.

Cells were grown to an optical density (OD) of 0.4 to 0.5 and treated with either 4 mM diamide or H_2_O for 20 min. Cells were then collected, washed, and lysed with glass beads in 5% *meta*-phosphoric acid. The culture supernatants were used for glutathione quantification by a glutathione (GSSG/GSH) detection kit (Enzo Life Science, Farmingdale, NY).

### Quantification of transcript levels by RT-qPCR.

Total RNA was extracted from cells using Trizol reagent (Invitrogen, Grand Island, NY) and chloroform extraction, followed by purification with RNeasy minicolumns (Qiagen, Redwood City, CA). RNA was reverse transcribed using an iScript cDNA synthesis kit (Bio-Rad, Des Plaines, IL). qPCR was performed with iTaq universal SYBR green supermix (Bio-Rad, Des Plaines, IL). Target gene transcript levels were normalized to transcript levels of elongation factor 1-alpha (*TEF1*).

### Data availability.

Plasmids pBV65 and pBV163 have been deposited in Addgene under no. 108363 and 108364, respectively.
